# Changes in antimicrobial resistance among Salmonella enterica Serovar typhimurium isolates from humans and cattle in the Northwestern United States, 1982-1997.

**DOI:** 10.3201/eid0506.990610

**Published:** 1999

**Authors:** M A Davis, D D Hancock, T E Besser, D H Rice, J M Gay, C Gay, L Gearhart, R DiGiacomo

**Affiliations:** Department of Veterinary Clinical Sciences, Washington State University, Pullman, WA 99164-6610, USA.

## Abstract

We compared antimicrobial resistance patterns of Salmonella enterica serovar Typhimurium (ST) of isolates from humans (n = 715) and cattle (n = 378) in the Pacific Northwest from 1982 through 1997. The major changes in antimicrobial resistance can be attributed to the widespread clonal dissemination of multidrug-resistant definitive phage type 104 ST.


### Changes in Antimicrobial Resistance among *Salmonella enterica* Serovar Typhimurium Isolates from Humans and Cattle in the Northwestern United States, 1982–1997

**Margaret A. Davis, Dale D. Hancock, Thomas E. Besser, Daniel H. Rice, John M. Gay, Clive Gay, Lynne Gearhart, and Ronald DiGiacomo**
Washington State University, Pullman, Washington, USA

We compared antimicrobial resistance patterns of *Salmonella enterica* serovar Typhimurium (ST) of isolates from humans (n = 715) and cattle (n = 378) in the Pacific Northwest from 1982 through 1997. The major changes in antimicrobial resistance can be attributed to the widespread clonal dissemination of multidrug-resistant definitive phage type 104 ST.

Enteritidis is the most frequent *Salmonella* serovar in all regions of the United States, except the Pacific Northwest where Typhimurium is the most frequent. In 1996, 31.1% of all *Salmonella* isolates from human sources in Washington, Oregon, and Idaho were serovar Typhimurium, while 14.6% were serovar Enteritidis (1). Typhimurium is also one of the most common *Salmonella* serovars from animal sources in the United States (2).

Use of antimicrobial drugs in food animals may lead to resistant strains of pathogens, which may be transmitted to humans through food (3,4). Although there is evidence that this transmission occurs, the contribution of antimicrobial use in food animals to resistance in bacteria infecting humans is the subject of debate (5-8).

We compared antimicrobial resistance patterns of *Salmonella enterica* serovar Typhimurium (ST) from human and cattle sources over a 15-year period and examined how these patterns relate to antimicrobial use in livestock and humans.

Address for correspondence: Margaret A. Davis, Field Disease Investigation Unit, Department of Veterinary Clinical Sciences, Washington State University, Pullman, WA 99164-6610, USA; fax: 509-335-0880; e-mail: madavis@vetmed.wsu.edu.

### The Study

We used all ST (including *S.* Typhimurium var Copenhagen) isolates from clinical bovine samples submitted to the Washington Animal Disease Diagnostic Laboratory from 1986 through 1997 and from cattle herds tested by the Field Disease Investigation Unit during salmonellosis outbreaks over the same period (n = 378). For herds sampled repeatedly over time, all but the first ST isolate (per year) were excluded. Antimicrobial resistance data were also available for ST isolates from cattle for 1982 through 1986 from clinical submissions to the Washington Animal Disease Diagnostic Laboratory. Isolates from human clinical specimens (n = 715) were obtained from the Washington State Department of Health Public Health Laboratory for 1989, 1994, 1996, and 1997, and from the Idaho Division of Health, Bureau of Laboratories for 1997.

Isolates from other laboratories were subcultured onto solid brain heart infusion agar. All isolates were maintained in a -70° C bank freezer in brain heart infusion broth containing 25% to 30% buffered glycerol. Isolates were streaked for isolation on sheep blood agar prior to susceptibility testing. Susceptibility testing for the antimicrobial drugs listed in Table 1 was done by a disk diffusion method (9) on

Table 1. Resistance to individual antibiotics among *Salmonella* Typhimurium isolates from cattle and humans

A. From cattle

| | Number (%) resistant by years | | | |
|---|---|---|---|---|
| | 1982-1985[a] | 1986-1990 | 1991-1994 | 1995-1997 |
| Antimicrobial drug | n = 49 | n = 116 | n = 90 | n = 123 |
| Ampicillin | 39 (79.6) | 99 (85.3) | 72 (80.0) | 113 (91.9) |
| Chloramphenicol[a] | 2 (4.1) | 2 (1.7) | 56 (62.2) | 90 (73.2) |
| Gentamicin | 1 (2.0) | 6 (5.2) | 14 (15.6) | 5 (4.1) |
| Kanamycin[a] | NA | 103 (90.4) | 49 (54.4) | 50 (40.7) |
| Streptomycin | 43 (87.8) | 109 (94.0) | 78 (86.7) | 116 (94.3) |
| Tetracycline | 43 (87.8) | 101 (87.1) | 77 (85.6) | 115 (93.5) |
| Trimethoprim | NA | 1 (0.9) | 11 (12.2) | 6 (4.9) |
| Trimethoprim-sulfamethoxazole | 7 (14.3) | 1 (0.9) | 11 (12.2) | 9 (7.3) |
| Triple sulfa | 39 (79.6) | 108 (93.1) | 75 (83.3) | 117 (95.1) |

B. From humans

| | Number (%) resistant by year | | | |
|---|---|---|---|---|
| | 1989 | 1994 | 1996 | 1997 |
| Antimicrobial drug | n = 90 | n = 189 | n = 187 | n = 249 |
| Ampicillin[a] | 22 (24.4) | 107 (56.6) | 109 (58.3) | 164 (65.9) |
| Chloramphenicol[a] | 3 (3.3) | 84 (44.4) | 92 (49.2) | 116 (46.6) |
| Gentamicin | 2 (2.2) | 5 (2.7) | 5 (2.7) | 2 (0.8) |
| Kanamycin | 25 (27.8) | 44 (23.3) | 34 (18.2) | 47 (18.9) |
| Streptomycin[a] | 42 (46.7) | 112 (59.3) | 109 (58.3) | 174 (69.9) |
| Tetracycline[a] | 36 (40.0) | 101 (53.4) | 109 (58.3) | 158 (63.5) |
| Trimethoprim | 0 | 7 (5.1) | 6 (3.1) | 5 (2.0) |
| Trimethoprim-sulfamethoxazole | 0 | 6 (3.7) | 6 (3.2) | 5 (2.0) |
| Triple sulfa[a] | 36 (40.0) | 123 (65.1) | 143 (76.5) | 231 (92.8) |

[a]Chi-square test for trend, p value <0.001.
NA, data not available.

Mueller-Hinton agar prepared according to National Committee for Clinical Laboratory Standards (NCCLS) guidelines (10,11). Ciprofloxacin susceptibility was tested on a subset of isolates systematically selected to include five per species per year.

Data for the analysis were divided into periods 1982-1986, 1987-1990, 1991-1994, and 1995-1997. These periods were chosen to compare isolates from cattle with isolates from humans for the years for which isolates from humans were available. Each isolate was classified as resistant or susceptible to each antimicrobial drug tested by the threshold zone size for resistance, as recommended by NCCLS (10,11). The proportions of isolates resistant to individual drugs and having each antimicrobial resistance pattern were computed by species and period. Significance testing of differences in proportions was done with Epi Info (12) using the chi-square test and the chi-square test for trend.

Marked changes in resistance to chloramphenicol were observed for isolates from both cattle and humans (Tables 1, 2). Before 1991, fewer than 5% of isolates from cattle and only 3% of the 1989 isolates from humans were resistant to chloramphenicol; by the mid-1990s, more than 70% (90 of 123) of isolates from cattle (p <0.01) and almost 50% (92 of 187) of isolates from humans (p <0.01) were resistant to chloramphenicol. Most (79%) isolates from cattle were resistant to ampicillin, streptomycin, tetracycline, and sulfonamides throughout the study period. Among isolates from humans, the proportion resistant to these drugs was significantly lower in 1989 than in 1997 (p <0.01). The proportion of isolates from cattle resistant to kanamycin was significantly less (p <0.01) in 1990 to 1994 than in 1986 to 1990. All isolates tested were susceptible to ciprofloxacin, and average zone sizes showed no evidence of decline during the period (data not shown).

Among isolates from both cattle and humans, the ACSSuT resistance pattern was the most

Table 2. Antimicrobial resistance patterns for *Salmonella* Typhimurium isolates from cattle and humans

A. Cattle

| | Number (%) | | | |
|---|---|---|---|---|
| | 1982-1985[a] n = 49 | 1986-1990 n = 114 | 1991-1994 n = 90 | 1995-1997 n = 123 |
| ACSSuT | 1 (2.0) | 1 (0.9) | 18 (20.0) | 55 (44.7) |
| ACKSSuT | | 0 | 25 (27.8) | 24 (19.5) |
| ASSuT | 24 (49.0) | 0 | 5 (5.6) | 6 (4.9) |
| AKSSuT | | 83 (72.8) | 4 (4.4) | 14 (11.4) |
| Su | 1 (2.0) | 1 (0.9) | 0 | 0 |
| All Others | 22 (45.0) | 24 (21.1) | 29 (32.2) | 20 (16.2) |
| Suscept[b] | 1 (2.0) | 5 (4.4) | 9 (10.0) | 4 (3.3) |

B. Humans

| | Number (%) | | |
|---|---|---|---|
| | 1989 n = 90 | 1994 n = 189 | 1996-1997 n = 436 |
| ACSSuT | 2 (2.2) | 44 (23.3) | 156 (35.8) |
| ACKSSuT | 0 | 26 (13.8) | 35 (8.0) |
| ASSuT | 0 | 14 (7.4) | 24 (5.5) |
| AKSSuT | 19 (21.1) | 2 (1.1) | 33 (7.6) |
| Su | 1 (1.1) | 9 (4.8) | 80 (18.4) |
| All Others | 25 (27.8) | 33 (17.5) | 47 (10.8) |
| Suscept[b] | 43 (47.8) | 61 (32.3) | 61 (14.0) |

[a]Data on kanamycin susceptibility not available for 1982-1985.
[b]Susceptible to all antimicrobial drugs tested.
A, ampicillin; C, chloramphenicol; T, tetracycline; G, gentamicin; K, kanamycin; S, streptomycin; Su, sulfonamide; Tmp, trimethoprim.

frequent and increased in frequency over the study period (Table 2). Before 1991, ACSSuT accounted for fewer than 4 (2%) of 255 of isolates from both species, and by the mid-1990s it accounted for more than 55 (40%) of 123 of isolates from cattle and more than 156 (35%) of 436 of isolates from humans. Isolates with the ACSSuT resistance pattern together with ACKSSuT-resistant isolates accounted for 79 (64%) of 123 isolates from cattle and 191 (44%) of 436 isolates from humans by the mid-1990s. As previously reported, 57 (95%) of 60 of these isolates were phage typed and found to be DT104 with a single pulsed-field gel electrophoresis pattern (13).

Isolates susceptible to all drugs tested were more common from human than cattle sources (166 [23.2%] of 715 vs. 19 [5.0%] of 378, $p < 0.01$). The proportion of isolates from humans susceptible to all drugs tested decreased substantially from 1989 to 1997 (chi-square test for trend, $p < 0.01$), while that of isolates from cattle was 10% or less for all periods studied (Table 2).

### Conclusions

Antimicrobial resistance has been commonly observed in human and bovine ST isolates since the earliest days of antimicrobial use. This study provides a longitudinal perspective on resistance in ST from cattle and humans in a region and allows insight as to the mechanism of changes in antimicrobial resistance. The greatest changes were in chloramphenicol and kanamycin resistance in isolates from cattle and ampicillin, chloramphenicol, streptomycin, sulfonamides, and tetracycline resistance in isolates from humans. Changes in resistance in isolates from both species were primarily due to the sharply increased occurrence of isolates displaying the ACSSuT resistance pattern, a reliable marker for multidrug-resistant definitive type 104 ST (MR-DT104) (14). This has been shown to be so in the Pacific Northwest through a subset of isolates from this study (13).

MR-DT104 was first detected almost simultaneously in several geographic areas, including the United Kingdom (15), the United States (13), and Canada (16). Molecular genetic studies indicate that the same gene cassette accounts for multiple resistance in isolates from these and other diverse geographic areas (17,18). This study not only provides supporting evidence that MR-DT104 from different regions are clonal in origin, but refutes the notion that the multiple antimicrobial resistance of this clone was due to acquisition of new resistance genes by indigenous ST in each region.

It is possible that local antimicrobial selection pressure played an important role in dissemination of MR-DT104 through cattle populations into the human population. However, several observations argue against this hypothesis. First, the rise in the percentage of resistance to chloramphenicol in isolates from cattle occurred after the withdrawal of the drug for use in food animals in the mid-1980s (19) and before the 1996 approval of florfenicol (a chloramphenicol analog that shares resistance loci with chloramphenicol in MR-DT104 [20]) for therapeutic use in cattle. Second, before the dissemination of MR-DT104, most isolates from cattle were resistant to ampicillin, streptomycin, sulfonamides, and tetracycline. It is not evident how, in the absence of chloramphenicol use, antimicrobial selection pressure would favor R-type ACSSuT over ASSuT, although it is possible that an unmeasured resistance factor favored the dissemination of MR-DT104 over ASSuT strains. Third, early in its global dissemination, MR-DT104 was isolated from several species of wildlife, which are not exposed to substantial amounts of antimicrobial drugs (13). Finally, reports of broad dissemination of *Salmonella* clones susceptible to antimicrobial drugs commonly used in livestock provide evidence that agent factors other than antimicrobial resistance are necessary for broad dissemination (21,22).

Nevertheless, some selection pressure that likely involved antimicrobial use must explain the high prevalence of antimicrobial resistance among ST. There is strong evidence that livestock are the main reservoir for human salmonellosis in industrialized countries (23); however, it would be an error to assume that the emergence of a globally disseminated clone can be attributed to antimicrobial use in livestock. A human reservoir exists for nontyphoidal *Salmonella*, including serovar Typhimurium, in developing countries (24,25), and there is strong evidence that antimicrobial use in humans has not only driven the emergence of multidrug-resistant clones in these regions but has resulted in an increasingly high prevalence of multiple resistance (26-29). Dissemination of multidrug-resistant *Salmonella* from developing countries, through human traffic, is well documented (30,31) and seems a more likely mode of international transport than the far more limited international livestock traffic.

Multidrug-resistant clones capable of global dissemination can emerge as a result of antimicrobial selection pressure in either livestock or humans; simply restricting antimicrobial use in livestock populations cannot prevent broad dissemination. The problem of globally distributed multidrug-resistant bacterial clones can be compared to the nosocomial scenario: prudent antimicrobial use is a sensible step, but the main effort must go toward preventing dissemination if the program is to be effective.

### Acknowledgments

The authors thank Jay Lewis, Donna Green, and Beth Mamer for their help in providing *Salmonella* isolates for this project.

Dr. Davis is a Ph.D. candidate in the Washington State University College of Veterinary Medicine Field Disease Investigation Unit. Formerly she was an epidemiologist at the Seattle-King County Department of Public Health. Dr. Davis is interested in the epidemiology and farm ecology of zoonotic enteric diseases, including *Escherichia coli* O157:H7 infection, salmonellosis, and campylobacteriosis.

### References

1. Centers for Disease Control and Prevention. U.S. Department of Health and Human Services. Salmonella surveillance. Annual Tabulation Summary 1996.
2. Ferris KE, Miller DA. Salmonella serotypes from animals and related sources reported during July 1996-June 1997. Proceedings of the 101st Annual Meeting of the United States Animal Health Association; 1997 Oct 18-24; Louisville, Kentucky. 1997. p. 423-43.
3. Oosterom J. Epidemiological studies and proposed preventive measures in the fight against human salmonellosis. Int J Food Microbiol 1991;12:41-51.
4. Khachatourians GG. Agricultural use of antibiotics and the evolution and transfer of antibiotic-resistant bacteria. CMAJ 1998;159:1129-36.
5. Piddock LJV. Does the use of antimicrobial agents in veterinary medicine and animal husbandry select antibiotic-resistant bacteria that infect man and compromise antimicrobial chemotherapy? J Antimicrob Chemother 1996;38:1-2.
6. Van den Bogaard AE. Antimicrobial resistance—relation to human and animal exposure to antibiotics. J Antimicrob Chemother 1997;40:453-4.
7. Vernon R. Ciprofloxacin-resistant *Salmonella typhimurium* DT104 [letter]. Vet Rec 1998;142:287.
8. Institute of Medicine. Antimicrobial resistance: Issues and options. Workshop report, Forum on Emerging Infections. Washington: National Academy Press; 1998.
9. Bauer AW, Kirby MMW, Sherris JC, Turck M. Antibiotic susceptibility testing by a standard single disk method. Am J Clin Pathol 1966;45:493-6.
10. National Committee for Clinical Laboratory Standards. Performance standards for antimicrobial disk susceptibility tests. 5th ed. Approved standard; M2-A5, Vol. 13, No. 24. Villanova (PA): The Committee; 1993.
11. National Committee for Clinical Laboratory Standards. Performance standards for antimicrobial disk susceptibility tests. 5th informational supplement; M100-S5, Vol. 14, No. 16. Villanova (PA): The Committee; 1994.
12. Dean AG, Dean JA, Coulombier D, Brendel KA, Smith DC, Burton AH, et al. Epi Info, Version 6: A word-processing, database, and statistics program for public health on IBM-compatible microcomputers. Atlanta (GA:) Centers for Disease Control and Prevention; 1995.
13. Besser TE, Gay CC, Gay JM, Hancock DD, Rice D, Pritchett LC, et al. Salmonellosis associated with *S. typhimurium* DT104 in the USA. Vet Rec 1997;140:75.
14. Glynn MK, Bopp C, Dewitt W, Dabney P, Mokhtar M, Angulo FJ. Emergence of multidrug-resistant *Salmonella enterica* serotype Typhimurium DT104 infections in the United States. N Engl J Med 1998;338:1333-8.

15. Threlfall EJ, Frost JA, Ward LR, Rowe B. Epidemic in cattle and humans of *Salmonella typhimurium* DT104 with chromosomally integrated multiple drug resistance. Vet Rec 1994;134:577.
16. Poppe C, Smart N, Khakhria R, Johnson W, Spika J, Prescott J. *Salmonella typhimurium* DT104: a virulent and drug-resistant pathogen. Can Vet J 1998;39:559-64.
17. Ridley A, Threlfall EJ. Molecular epidemiology of antibiotic resistance genes in multiresistant epidemic *Salmonella typhimurium* DT104. Microb Drug Resist 1998;4:113-8.
18. Casin I, Breuil J, Brisabois A, Moury F, Grimont F, Collatz E. Multidrug-resistant human and animal *Salmonella typhimurium* isolates in France belong predominantly to a DT104 clone with the chromosome- and integron-encoded ß-lactamase PSE-1. J Infect Dis 1999;179:1173-82.
19. Knapp WA Jr. Report of the Committee on Pharmaceuticals, Pesticides, and Related Toxicology. Proceedings of the 88th Annual Meeting of the United States Animal Health Association. 1984 Oct 21-26; Fort Worth, Texas.
20. Bolton LF, Kelley LC, Lee MD, Fedorka-Cray PJ, Maurer JJ. Detection of multidrug-resistant *Salmonella enterica* serotype *typhimurium* DT104 based on a gene which confers cross-resistance to florfenicol and chloramphenicol. J Clin Microbiol 1999;37:1348-51.
21. Khakhria R, Bezanson G, Duck D, Lior H. The epidemic spread of *Salmonella typhimurium* phage type 10 in Canada (1970-1979). Can J Microbiol 1983;29:1583-8.
22. Passaro DJ, Reporter R, Mascola L, Kilman L, Malcolm GB, Rolka H, et al. Epidemic *Salmonella enteritidis* infection in Los Angeles County, California—the predominance of phage type 4. West J Med 1996;165:126-30.
23. Hancock DD, Lynn TV, Besser TE, Wikse SE. Feasibility of preharvest food safety control. Compendium on Continuing Education for the Practicing Veterinarian 1997;19:S200-7.
24. Gracey M, Iveson JB, Sunoto, Suharyono. Human salmonella carriers in a tropical urban environment. Trans R Soc Trop Med Hyg 1980;74:479-82.
25. Vahaboglu H, Dodanli S, Eroglu C, Öztürk R, Soyletir G, Yildirim I, et al. Characterization of multiple-antibiotic-resistant *Salmonella typhimurium* strains: molecular epidemiology of PER-1-producing isolates and evidence for nosocomial plasmid exchange by a clone. J Clin Microbiol 1996;34:2942-6.
26. Kariuki S, Gilks C, Corkill J, Kimari J, Benea AP, Hart CA. Multi-drug resistant non-typhi salmonellae in Kenya. J Antimicrob Chemother 1996;38:425-34.
27. Agarwal KC, Garg RK, Panhotra BR, Verma AD, Ayyagari A, Mahanta J. Drug resistance in Salmonellae isolated at Chandigarh (India) during 1972-1978. Antonie Van Leeuwenhoek 1980;46:383-90.
28. Sharma KB, Bhat MB, Pasricha A, Vaze S. Multiple antibiotic resistance among salmonellae in India. J Antimicrob Chemother 1979;5:15.
29. Ling J, Chau PY, Rowe B. Salmonella serotypes and incidence of multiply-resistant salmonellae isolated from diarrhoeal patients in Hong Kong from 1973-1982. Epidemiol Infect 1987;99:295-306.
30. Centers for Disease Control. Multiresistant *Salmonella* and other infections in adopted infants from India. MMWR Morb Mortal Wkly Rep 1982;31:285-7.
31. Seyfarth AM, Wegener HC, Frimodt-Møller. Antimicrobial resistance in *Salmonella enterica* subspecies *enterica* serovar *typhimurium* from humans and production animals. J Antimicrob Chemother 1997;40:67-75.

